# Genome-wide association study of cognitive functions and educational attainment in UK Biobank (*N*=112 151)

**DOI:** 10.1038/mp.2016.45

**Published:** 2016-04-05

**Authors:** G Davies, R E Marioni, D C Liewald, W D Hill, S P Hagenaars, S E Harris, S J Ritchie, M Luciano, C Fawns-Ritchie, D Lyall, B Cullen, S R Cox, C Hayward, D J Porteous, J Evans, A M McIntosh, J Gallacher, N Craddock, J P Pell, D J Smith, C R Gale, I J Deary

**Affiliations:** 1 Centre for Cognitive Ageing and Cognitive Epidemiology, University of Edinburgh, Edinburgh, UK; 2 Department of Psychology, University of Edinburgh, Edinburgh, UK; 3 Medical Genetics Section, University of Edinburgh Centre for Genomic and Experimental Medicine and MRC Institute of Genetics and Molecular Medicine, Edinburgh, UK; 4 Queensland Brain Institute, The University of Queensland, Brisbane, QLD, Australia; 5 Division of Psychiatry, University of Edinburgh, Edinburgh, UK; 6 Institute of Health and Wellbeing, University of Glasgow, Glasgow, UK; 7 MRC Human Genetics Unit, MRC Institute of Genetics and Molecular Medicine, University of Edinburgh, Edinburgh, UK; 8 Department of Psychiatry, University of Oxford, Oxford, UK; 9 Institute of Psychological Medicine and Clinical Neurosciences, MRC Centre for Neuropsychiatric Genetics and Genomics, Cardiff University, Cardiff, UK; 10 MRC Lifecourse Epidemiology Unit, University of Southampton, Southampton, UK

## Abstract

People's differences in cognitive functions are partly heritable and are associated with important life outcomes. Previous genome-wide association (GWA) studies of cognitive functions have found evidence for polygenic effects yet, to date, there are few replicated genetic associations. Here we use data from the UK Biobank sample to investigate the genetic contributions to variation in tests of three cognitive functions and in educational attainment. GWA analyses were performed for verbal–numerical reasoning (*N*=36 035), memory (*N*=112 067), reaction time (*N*=111 483) and for the attainment of a college or a university degree (*N*=111 114). We report genome-wide significant single-nucleotide polymorphism (SNP)-based associations in 20 genomic regions, and significant gene-based findings in 46 regions. These include findings in the *ATXN2*, *CYP2DG*, *APBA1* and *CADM2* genes. We report replication of these hits in published GWA studies of cognitive function, educational attainment and childhood intelligence. There is also replication, in UK Biobank, of SNP hits reported previously in GWA studies of educational attainment and cognitive function. GCTA-GREML analyses, using common SNPs (minor allele frequency>0.01), indicated significant SNP-based heritabilities of 31% (s.e.m.=1.8%) for verbal–numerical reasoning, 5% (s.e.m.=0.6%) for memory, 11% (s.e.m.=0.6%) for reaction time and 21% (s.e.m.=0.6%) for educational attainment. Polygenic score analyses indicate that up to 5% of the variance in cognitive test scores can be predicted in an independent cohort. The genomic regions identified include several novel loci, some of which have been associated with intracranial volume, neurodegeneration, Alzheimer's disease and schizophrenia.

## Introduction

Cognitive functions have important roles in human mental and physical well-being. Better cognitive function in youth is associated with lower risk of some psychiatric disorders and physical illness later in the life course, and with reduced mortality risk.^1, 2^ The reverse is also true; some mental and physical illnesses are associated with a lowering of cognitive capabilities in youth and over the life course.^3, 4^ Higher cognitive ability in youth is associated also with higher educational attainment and adult social position.^5^ Domains of cognitive functioning differ in their associations with ageing; some have trajectories of decline (for example, processing speed and some types of memory), whereas others (for example, knowledge-based tests) hold their levels better over the adult life course.^6, 7^ Therefore, it is important to understand the causes of people's differences in cognitive functions.

One source of cognitive differences is genetic variation. Cognitive functions have a substantial heritability. This has been found by using twin and family studies,^8, 9, 10, 11, 12^ and by molecular genetic methods, such as Genome-wide Complex Trait Analysis (GCTA-GREML),^13, 14^ which estimates heritability based on common single-nucleotide polymorphisms (SNPs).

Some explanation is required regarding cognitive phenotypes. All tests of cognitive ability correlate positively, though not perfectly; that is, people who do well on one type of cognitive test tend to do well on the others.^15^ It is this regularity that is the basis for the construct of general cognitive ability, which is usually abbreviated to *g*. There are also separable domains of cognitive functioning.^10, 15^ Differences in individual cognitive test score performances may be due to: (1) differences in general cognitive function described by the variance shared by all cognitive domains, *g*; (2) differences in test performance specific to a cognitive domain; and (3) differences specific to a particular test.^16^ Twin and SNP-based GCTA-GREML studies have found that there is substantial heritability for general cognitive function, and also some heritability for cognitive domains and specific cognitive skills.^17, 18^ They also find that there are significant genetic correlations among tests of different cognitive domains, and between cognitive abilities and education, which also shows substantial heritability.^18, 19^

Genome-wide association studies (GWAS) of cognitive functions have been successful in estimating SNP-based heritability, and in using summary GWAS data to make predictions of cognitive phenotypes in independent samples.^13^ However, they have been less successful in identifying the specific genetic variants that cause cognitive differences. The largest studies to date have been the CHARGE-Cognitive Working Group's studies^13, 20, 21^ and those on educational phenotypes by the Social Science Genetics Association Consortium.^22, 23, 24^ In a study of 53 949 individuals with data on general cognitive function, there were three genome-wide significant hits in three genomic regions, with the closest genes being *APOE/TOMM40*, *AKAP6* and *MIR2113*.^13^ In a study of 32 070 individuals with data on processing speed (mostly digit-symbol substitution-type tests) there was one genome-wide significant hit, near *CADM2*.^21^ In a study of 29 076 individuals with data on verbal declarative memory there were three genome-wide significant hits, near *APOE* and genes associated with immune response.^20^

Given the large phenotypic and genetic correlations between general cognitive ability and educational attainment,^18, 25, 26^ others have used the latter as a proxy phenotype for cognitive ability.^23^ This has led to finding three independent genome-wide significant variants, with the nearest genes being *LOC100129185*, *LRRN2* and *LOC150577*.^23, 24^ The latter two hits were with the phenotype of having attained a college or university degree, which is the educational phenotype used in the present study. Genome-wide SNPs from GWAS analyses of education phenotypes identified variants related to cognitive performance phenotypes, implicating the genes *KNCMA1*, *NRXN1*, *POU2F3* and *SCRT*.^23^ There has been replication of a SNP (rs1906252) that influences both education and general cognitive function.^13, 27^

GWAS meta-analytic studies of cognitive functions have been relatively unsuccessful in finding specific genetic variants that influence cognitive phenotypes, principally because the numbers of subjects are too small. The study of other complex phenotypes such as height suggests that many variants will be found as participant numbers increase to and beyond six figures.^28^ Second, the cognitive GWAS consortia studies to date have used several different assessments to represent each cognitive construct across different samples, and this may have led to phenotypic heterogeneity in the derived measures.^13, 20, 21, 27^ Third, studies to date have used samples whose genotyping has been carried out in different centres with different arrays and different quality control (QC) procedures.^13, 20, 21, 27^ Fourth, studies have tended to examine one cognitive phenotype or domain in isolation.^13, 20, 21, 27^

The present study directly addresses the limitations of previous molecular genetic studies of cognitive functions. It presents genome-wide association analyses of reasoning, processing speed, declarative memory, and educational attainment in the UK Biobank sample. The number of subjects is over 100 000 for most analyses. All participants took the same cognitive tests with the same instructions. All participants included in the current analysis were of white British ancestry. Genotyping was also standardised across the same arrays and QC procedures. The study addresses three important cognitive domains and educational attainment in a single report. These advantages are likely contributors to the relative success in finding many new genetic variants associated with cognitive functions.

## Materials and Methods

This study includes baseline data from the UK Biobank Study (http://www.ukbiobank.ac.uk).^29^ UK Biobank received ethical approval from the Research Ethics Committee. The REC reference for UK Biobank is 11/NW/0382. The present analyses were conducted under UK Biobank data application numbers 10279 and 7898.

### Participants

The UK Biobank is a health research resource that aims to improve the prevention, diagnosis and treatment of a wide range of illnesses. Between the years 2006 and 2010, about 500 000 people aged from middle age to older age were recruited from across Great Britain. Data were collected on cognitive functions, physical and mental health, lifestyle, socio-demographic information, food intake and family medical history. For the present study, 112 151 community-dwelling individuals (58 914 females, 53 237 males) aged 40–73 years (mean=56.91 years, s.d.=7.93) with genome-wide genotyping were available.

### Cognitive assessment

#### Verbal–numerical reasoning

Verbal–numerical reasoning was measured using a 13-item test presented on a touchscreen computer. The test included six verbal and seven numerical questions, all with multiple-choice answers, and had a time limit of two minutes in total. An example verbal item is: ‘If Truda's mother's brother is Tim's sister's father, what relation is Truda to Tim?' (possible answers: ‘aunt/sister/niece/cousin/no relation/do not know/prefer not to answer'). An example numerical item is: ‘If 60 is more than half of 75, multiply 23 by 3. If not subtract 15 from 85' (possible answers: ‘68/69/70/71/72/do not know/prefer not to answer'). The verbal–numerical reasoning score was the total score out of 13. The Cronbach *α-*coefficient for the 13 items was 0.62.

#### Reaction time

Reaction time (RT) was measured using a computerized ‘Snap' game. Participants were shown cards with symbols on a computer screen, and were directed to push a button on a nearby button box as quickly as possible with their dominant hand if the two cards had matching symbols. There were four practice trials to begin, followed by eight experimental trials, of which four had matching symbols. Each participant's RT score was their mean time (in milliseconds) to press the button for these four matching trials. The reliability (internal consistency) of these trials, using Cronbach's *α*, was 0.85. Before analysis, one participant with an outlying score was removed, and the data were log-transformed.

#### Memory

Memory was measured using a ‘pairs matching' task on a touchscreen computer. Participants observed a randomly arranged grid of 12 ‘cards' with six pairs of matching symbols for 5 s. The symbols were then hidden, and the participant was instructed to select, from memory, the pairs that matched, in the fewest possible number of attempts. Responses were made by touching consecutive pairs on the screen. No time limit was imposed; participants were free to make as many attempts as necessary for them to correctly match all the pairs. The memory score was the total number of errors made during this task. The test was preceded by a simpler, three-pair practice version. A log+1 transformation was applied to the memory variable before analysis.

For all three cognitive tests, repeated measurements were available on a subset of participants (Table 1). The mean time difference between baseline and the repeat testing was 4.3 years (s.d. 0.9), with a range of 2.1–7.0 years. The test–retest correlations were 0.65 for verbal–numerical reasoning (*n*=4 696), 0.54 for RT (*n*=20 188) and 0.15 for memory (*n*=19 872).

#### Educational attainment

To measure educational attainment, participants were asked, 'which of the following qualifications do you have? (You can select more than one)'. Possible answers were: ‘college or university degree/A levels or AS levels or equivalent/O levels or GCSE or equivalent/CSEs or equivalent/NVQ or HND or HNC or equivalent/Other professional qualifications, for example, nursing, teaching/none of the above/prefer not to answer'. We created a binary education variable indexing whether or not each participant had attained a college or university-level degree. This follows previous studies that have used similar binary variables in GWAS studies as a successful proxy for cognitive function.^23^

### Genotyping and quality control

152 729 UK Biobank samples were genotyped using either the UK Bileve (*N*=49 979) or the UK Biobank axiom array (*N*=102 750). Genotyping was performed on 33 batches of ~4700 samples by Affymetrix (High Wycombe, UK). Initial QC of the genotyping data was also performed by Affymetrix. Further details are available of the sample processing specific to the UK Biobank project (http://biobank.ctsu.ox.ac.uk/crystal/refer.cgi?id=155583) and the Axiom array (http://media.affymetrix.com/support/downloads/manuals/axiom_2_assay_auto_workflow_user_guide.pdf). Before the release of the UK Biobank genetic data a stringent QC protocol was applied, which was performed at the Wellcome Trust Centre for Human Genetics. Details of this process can be found here (http://biobank.ctsu.ox.ac.uk/crystal/refer.cgi?id=155580).

Before the analyses described below, further QC measures were applied. Individuals were removed sequentially based on non-British ancestry (within those who self-identified as being British, principal component analysis was used to remove outliers, *n*=32 484), high missingness (*n*=0), relatedness (*n*=7,948), QC failure in UK Bileve (*n*=187), and gender mismatch (*n*=0). A sample of 112 151 individuals remained for further analyses.

#### Imputation

An imputed data set was made available in which the UK Biobank interim release was imputed to a reference set combining the UK10K haplotype and 1000 Genomes Phase 3 reference panels. Further details can be found at the following URL: http://biobank.ctsu.ox.ac.uk/crystal/refer.cgi?id=157020. The association analyses were restricted to autosomal variants with a minor allele frequency >0.1% and an imputation quality score of 0.1 or greater (*N*~17.3 m SNPs).

### Statistical analyses

All phenotypes were adjusted for age, gender, assessment centre, genotyping batch, genotyping array and 10 principal components to correct for population stratification before all analyses. For RT, 111 483 individuals remained for further analyses. For memory, 112 067 individuals remained for further analyses. The verbal–numerical reasoning test was added to the cognitive battery part-way through the study and was performed on a subset of 36 035 individuals who also had genotyping. For Educational Attainment, 111 114 individuals were available for further analyses.

#### Association analyses

Genotype–phenotype association analyses were performed on the imputed data set using SNPTest v2.5.1.^30^ SNPTEST v.2.5.1 can be found at the following URL: https://mathgen.stats.ox.ac.uk/genetics_software/snptest/snptest.html#introduction). An additive model was specified using the ‘frequentist 1' option. To account for genotype uncertainty, we analysed the genotype dosage scores.

To determine the number of independent signals from the genotype–phenotype analyses, LD clumping was used.^31, 32^ The method was applied to the GWAS output for each phenotype separately, using the 1000 genomes to provide a measure of LD between the SNPs.^33^ SNPs were selected for the analysis if they attained a genome-wide significant (*P*<5 × 10^−^^8^) association with the respective phenotype. Next, SNPs in LD of *r*^2^>0.1 and within 500 kb of the index SNP were included in the clump. SNPs from within this region were assigned to the clump if they had a *P*-value<1 × 10^−5^.

Gene-based association analyses were performed using MAGMA.^34^ The gene-based statistics were derived using the results of the GWA analyses conducted on each phenotype. Genetic variants were assigned to genes based on their position according to the NCBI 37.3 build with no additional boundary placed around the genes; this resulted in a total of 18 062 genes being analysed. The European panel of the 1000 Genomes data (phase 1, release 3) was used as a reference panel to account for linkage disequilibrium.^33^ A genome-wide significance threshold for gene-based associations was calculated using the Bonferroni method (*α*=0.05/18 062; *P*<2.8 × 10^−^^6^).

Lookups were performed of the genome-wide significant SNP-based findings for the four UK Biobank traits in already-published GWAS of general cognitive function^13^, educational attainment^24^ (years of education and college degree) and childhood intelligence.^35^ For general cognitive function^13^ only a subset of the published data was available due to individual cohort restrictions on data usage (*N*=36 840; see Supplementary Methods). We also investigated replication of published genome-wide significant findings in the present study, by comparing the SNP and gene-based association results to published findings for educational attainment (SSGAC),^23, 24^ general cognitive function (CHARGE-cognitive),^13^ memory (CHARGE-cognitive),^20^ processing speed/executive function (CHARGE-cognitive),^21^ Alzheimer's disease (I-GAP),^36^ intracranial volume (representing brain size)^37^ and childhood intelligence.^35^

#### Estimation of SNP-based heritability and genetic correlations

Univariate GCTA-GREML^38^ analyses were used to estimate the proportion of variance explained by all common SNPs for each of the cognitive phenotypes and educational attainment. A relatedness cutoff of 0.025 was used in the generation of the genetic relationship matrix. LD score regression analyses were used to derive genetic correlations among the cognitive phenotypes, and between them and educational attainment. We followed the data processing pipeline devised by Bulik-Sullivan *et al.*^39^

#### Polygenic prediction

Polygenic profile scores were created using PRSice^40^ for the UK Biobank cognitive phenotypes and educational attainment in genotyped participants of Generation Scotland's Scottish Family Health Study (GS, *n*=19 994);^18, 41^ and the Lothian Birth Cohort of 1936 (LBC1936, *n*=1005).^14, 42^ Individuals were removed if they had contributed to both GS and UK Biobank. Polygenic profiles are the summation of an individual's genotype across many genetic loci, weighted by the effect size estimated from a GWAS on the trait of interest. SNPs with a minor allele frequency <0.01 were removed before creating polygenic profile scores. Genotypes were LD pruned using clumping to obtain SNPs in linkage equilibrium with an *r*^2^<0.25 within a 200 bp window. Five polygenic profile scores were created for all four phenotypes containing SNPs according to the significance of their association with the phenotype, at *P*-value cutoffs of 0.01, 0.05, 0.1, 0.5 and all SNPs from the original GWAS. Linear regression models were used to examine the associations between the polygenic profiles for the UK Biobank cognitive variables and the target phenotypes in GS and LBC1936, which included multiple measures of cognitive ability, adjusted for age at measurement, sex and the first five (GS) or four (LBC1936) genetic principal components for population stratification. All models were corrected for multiple testing across all polygenic profile scores at all five thresholds in each cohort using the false discovery rate method.^43^

#### Pathway and functional genomic analyses

Pathway and functional genomic analyses were performed using the GWA results for each of the cognitive phenotypes. These included DEPICT analyses^44^ and reference to Regulome DB^45^ (http://www.regulomedb.org/) and the Genotype-Tissue Expression Portal (http://www.gtexportal.org). Further details of these methods can be found in Supplementary Methods.

## Results

A description of the UK Biobank cohort is presented in Supplementary Table S1. Just under one-third of the sample (*n*=33 852, 30.5%) had a college or university degree. When scored such that higher scores represented better performance, the phenotypic correlations between the cognitive tests were all positive (Table 2). Verbal–numerical reasoning correlated with RT and memory at *r*=0.16 and 0.18, respectively. RT correlated with memory at *r*=0.12. The point-biserial correlations of educational attainment with verbal–numerical reasoning, RT, and memory were *r*=0.34, 0.10 and 0.05, respectively.

**Figure 1:**
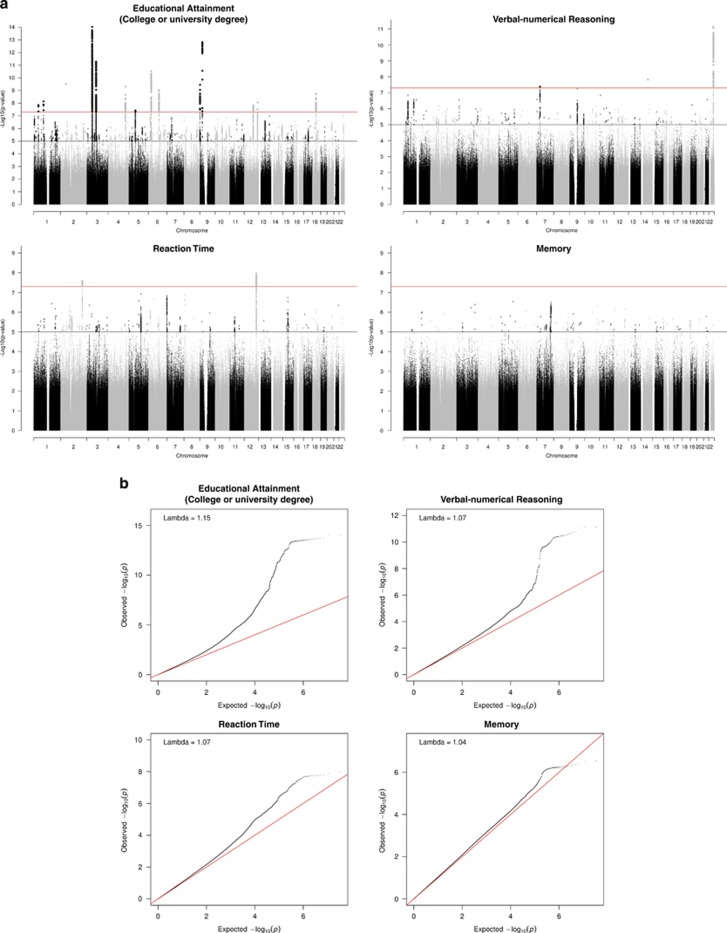
(**a**) Manhattan and (**b**) Q–Q plots of *P*-values of the SNP-based association analyses for each of the cognitive phenotypes: educational attainment, verbal–numerical reasoning, reaction time and memory. The red line indicates the threshold for genome-wide significance (*P*<5 × 10^−8^); the grey line indicates the threshold for suggestive significance (*P*<1 × 10^−^^5^). SNP, single-nucleotide polymorphism.

The results of the GWAS analyses are presented below; Manhattan and QQ Plots for each trait are shown in Figure 1.

### Verbal–numerical reasoning

A total of 149 SNPs from three genomic regions were significantly associated with verbal–numerical reasoning scores (Supplementary Table S2). Three independent signals were identified. The strongest signal was on chromosome 22 in a region that includes: the cytochrome P450 gene *CYP2D6*, which is linked to drug metabolism; NADH dehydrogenase (ubiquinone) 1 alpha subcomplex, 6, 14 kDa (*NDUFA6*), which is involved in mitochondrial function;^46^ and septin 3 (*SEPT3*), which is associated with Alzheimer's disease.^47^ The two other regions included: SNPs in phosphodiesterase 1C, calmodulin-dependent 70 kDa (*PDE1C*), a calmodulin-dependent PDE that is stimulated by a calcium-calmodulin complex;^48^ and a single SNP in Fucosyltransferase 8 (alpha (1,6) fucosyltransferase (*FUT8*), which catalyses the transfer of fucose from GDP-fucose to N-linked type complex glycopeptides.^49^ These were on chromosomes 7 and 14, respectively.

The gene-based analysis of verbal–numerical reasoning identified 17 significant genes from across seven regions, including multiple hits on chromosome 22, such as *SEPT3*, *CYP2D6* and *NDUFA6* (Supplementary Table S3). Other gene-based hits linked to neurobiological pathways include: Ataxin2-like (*ATXN2L*) on chromosome 16 (a member of the spinocerebellar ataxia family which is associated with neurodegenerative disorders); amyloid beta (A4) Precursor Protein-Binding, Family A, Member 1(*APBA1*) on chromosome 9, which interacts with the Alzheimer's disease amyloid precursor protein;^50^ and SH2B Adaptor Protein 1 (*SH2B1*) on chromosome 16, previously associated with type 2 diabetes.^51^

The proportion of variance in verbal–numerical reasoning that was explained by all common genetic variants was 31% (GCTA-GREML estimate 0.31, s.e.m.=0.018) (Table 1).

### Reaction time

For RT there were 36 SNP hits at the genome-wide significance threshold spanning two regions, one on chromosome 2 and the other on chromosome 12 (Supplementary Table S2). Two independent signals were identified from these top hits, including: a variant in the SH2B Adaptor Protein 3 (*SH2B3*) gene on chromosome 12, which is involved in signalling activities by growth factor and cytokine receptors; and a variant in spermatogenesis-associated, serine-rich 2-like (*SPATS2L*) (on chromosome 2). The chromosome 12 locus has previously been linked to a wide spectrum of disease susceptibilities affecting multiple organs, including neurodegenerative conditions and longevity.^52^

In the gene-based analysis, 23 genes from across nine regions were identified as having significant associations with RT (Supplementary Table S3). These included: *SH2B3* and Ataxin2 (*ATXN2*), associated with spinocerebellar ataxia 2^53^ on chromosome 12; autophagy/beclin-1 regulator 1 (*AMBRA1*), important in autophagy and the development of the nervous system^54^ on chromosome 11; diacylglycerol kinase, zeta (*DGKZ*), involved in intracellular signalling,^55^ on chromosome 5; and neuron navigator 1 (*NAV1*), expressed in the nervous system and thought to have a role in neuronal development and regeneration,^56^ on chromosome 1.

The GCTA-GREML estimate showed that 11% of the variance in RT can be explained by common genetic variants (estimate 0.11, s.e.m.=0.006) (Table 1).

### Memory

There were no genome-wide significant SNP-based findings for memory scores, despite the sample size being as large as for both RT and educational attainment and three times larger than that available for verbal–numerical reasoning. Two gene-based results from separate regions were identified for memory (Supplementary Table S3). These were: the exocyst complex component 4 (*EXOC4*), a component of the exocyst complex which is required for targeting exocytic vesicles to specific docking sites on the plasma membrane and has been associated with rate of cognitive decline in Alzheimer's disease;^57^ and exostosin glycosyltransferase 1 (*EXT1*), associated with type I multiple exostoses,^58^ on chromosomes 7 and 8 respectively.

The SNP-based GCTA-GREML estimate was 0.05 (s.e.m.=0.006), that is, 5% of the variance in memory test scores can be explained by common genetic variants (Table 1).

### Educational attainment

There were 1 115 SNPs that were associated with educational attainment at the genome-wide significance threshold (*P*<5 × 10^−8^) (Supplementary Table S2). The most significant was on chromosome 3 in a gene-dense region with more than 30 different genes. The top hit, in the Ras homologue family member A (*RHOA*) gene is an insertion/deletion and therefore was not included in the LD clumping analysis. After LD clumping, there were 15 independent signals, including: four on chromosome 3, two of which were in the cell adhesion molecule 2 (*CADM2*) gene, which is involved in synapse organization;^59^ and one in the CaM kinase-like vesicle-associated (*CAMKV*) gene.

A gene-based analysis identified 95 genes from across 28 regions that were significantly associated with Educational Attainment (Supplementary Table S3). The top hit from the gene-based analysis was MON1 secretory trafficking family member A (*MON1A*), involved in membrane trafficking,^60^ which is on chromosome 3. The *CADM2* gene was also significant. *ATXN2L* and *SH2B1*, which were significant in the verbal–numerical reasoning gene-based analysis, were also significantly associated with educational attainment.

The common genetic variants from the genotyped SNPs explained 21% of the variance in educational attainment (GCTA-GREML estimate 0.21, s.e.m.=0.006) (Table 1).

### Polygenic profile scoring

**Figure 2:**
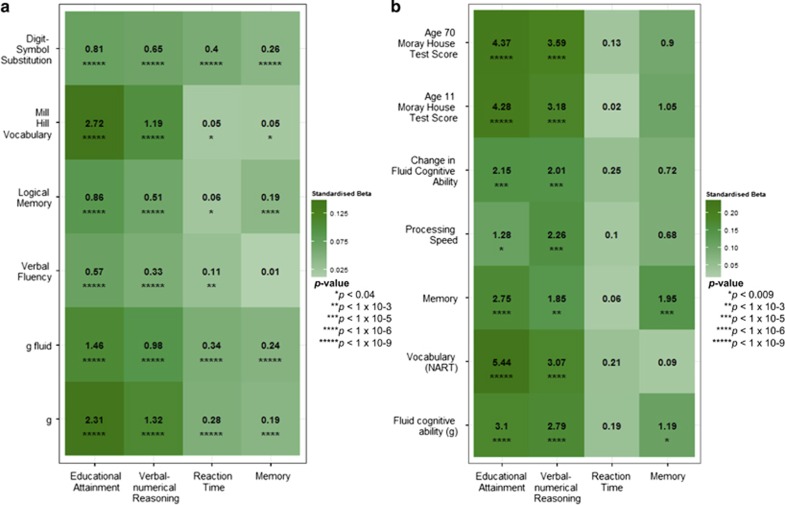
Heat maps of associations between the polygenic profile scores for cognitive phenotypes in UK Biobank and cognitive ability in Generation Scotland (**a**) and the Lothian Birth Cohort 1936 (**b**). Stronger associations are indicated by darker shades. The amount of variance (%) explained is indicated for each association. Further information can be found in Supplementary Table 4.

The GWAS results for the three cognitive tests and the educational attainment measure were used to build polygenic profile scores in two independent cohorts, the Lothian Birth Cohort 1936 (LBC1936) and GS. Significant predictions were observed across almost all thresholds for all of the predictors for the cognitive phenotypes measured in LBC1936 and GS (Figure 2 and Supplementary Table S4). The largest proportion of variance explained in LBC1936 was 5.4% for a vocabulary-based test (the National Adult Reading Test)^61^ using the Educational Attainment polygenic score with a SNP inclusion threshold of all SNPs from the GWAS. In GS the best prediction was also for the vocabulary measure; 2.8% of the variance was explained, again by the educational attainment polygenic score, and with a SNP inclusion threshold of *P*<0.50.

### Genetic correlations

LD score regression was used to estimate the genetic correlations between the three cognitive traits and educational attainment. When scored such that higher scores represented better performance, the genetic correlations were all positive (Table 2). The largest genetic correlations were observed between verbal–numerical reasoning and educational attainment (*r*_g_=0.73, s.e.m.=0.04), memory (*r*_g_=0.44, s.e.m.=0.06) and RT (r_g_ 0.21 s.e.m.=0.05). These correlations have been published previously.^62^

### Replication of SNP- and gene-based hits

We sought replication of the top SNP-based findings in published GWAS of general cognitive function,^13^ educational attainment^24^ (years of education and college degree), and childhood cognitive function^35^ (Supplementary Table S5). We also compared SNP- and gene-based hits (where available) from the previous literature with the corresponding results from the present UK Biobank analysis (Supplementary Tables S6 and S7).

We first describe the SNP-based lookup in the already-published GWAS (Supplementary Table S5). We present lookups for all available SNPs, as not all SNPs from the current study were available due to differences in imputation reference panels. We find that, of the 1115 genome-wide significant SNPs associated with educational attainment, 327 (general cognitive function), 326 (years of education), 326 (college degree) and 267 (childhood intelligence) were available in the published GWAS. Of these 158, 240, 211 and 47, respectively, showed replication at *P*<0.05, for general cognitive function, years of education, college degree and childhood cognitive function. For verbal–numerical reasoning we report 149 SNPs reaching genome-wide significance; 37 were available for general cognitive function, years of education and college degree, and 29 for childhood intelligence. Thirty-six SNPs in the chromosome 22 region replicate for general cognitive function, one SNP on chromosome 7 demonstrated replication with childhood intelligence, and no replication was observed with either years of education or college degree. None of the 36 SNPs associated with RT replicated in any of the above-listed published GWAS.

The SNP-based lookups, within this new UK Biobank sample, of previously reported genome-wide significant findings are detailed in Supplementary Table S6. Replication is reported where *P*<0.05 in the UK Biobank GWASs. For the 13 genome-wide significant SNPs in the 2015 GWAS of general cognitive function,^13^ 11 SNPs in the chromosome 6 region were replicated for educational attainment, and verbal–numerical reasoning. A single SNP on chromosome 14 (rs17522122 in the *AKAP6* gene) was replicated in verbal–numerical reasoning, RT, and memory. Of the three memory hits reported by Debette et al.,^20^ one SNP (rs4420638 in the *APOC1* gene) replicated in the UK Biobank memory GWAS.

Of the three educational attainment SNP hits (two for college degree, rs11584700, rs4851266; one for years of education, rs9320913) reported by Rietveld *et al.*,^24^ all three replicated in UK Biobank for educational attainment, and two SNPs, rs4851266 and rs9320913, also replicated for verbal–numerical reasoning. Of the three SNPs associated with educational attainment and cognitive function (rs1487441, rs7923609 and rs2721173) in Rietveld *et al.*,^23^ all demonstrated replication with educational attainment in UK Biobank, rs1487441 and rs2721173 with verbal–numerical reasoning, and rs2721173 with RT.

Of the 21 genome-wide significant hits reported in the most recent Alzheimer's disease GWAS, one SNP, rs983392, showed replication with memory in UK Biobank.

One SNP, rs17689882, in the *CRHR1* gene that was associated with intracranial volume^37^ was replicated in UK Biobank with both educational attainment and verbal–numerical reasoning.

For the gene-based lookups, the *HMGN1* gene reported in the general cognitive function paper^13^ did not replicate for any of the traits (Supplementary Table S7). The *FNBP1L* gene, which has been associated with childhood cognitive ability,^35^ was replicated in the educational attainment analysis (*P*=0.004). Of the seven gene-based hits for educational attainment^24^ (college versus no college), two (*PIK3C2B* and *TET2*) were replicated in UK Biobank for the educational attainment variable. None of the genes was replicated for the other three traits. For the years of education variable examined by Rietveld *et al.*,^24^ 12 out of the 16 significant genes were replicated in the UK Biobank educational attainment analysis. Five of these genes also replicated for verbal–numerical reasoning. There was no overlap for the RT or memory traits. Of the genes linked to educational attainment and cognitive function in Rietveld et al.,^23^ *NRXN1* was associated with verbal–numerical reasoning and *POU3F2* with memory in UK Biobank.

### DEPICT results

For the educational attainment phenotype, gene prioritization as implemented in DEPICT indicated a role for 28 genes at false discovery rate<0.05 (Supplementary Table S8). No genes showed statistically significant links to verbal–numerical reasoning, RT or memory. In the gene-set analyses, two gene sets were significantly enriched for verbal–numerical reasoning; these were ‘regulation of cell morphogenesis' (GO:0022604), false discovery rate ⩽0.01, and ‘CLTC PPI subnetwork' (ENSG00000141367), false discovery rate ⩽0.05. No significant results were found with educational attainment, RT or memory.

The tissue enrichment analyses yielded no significant findings for any of the cognitive function phenotypes. However, nominally significant results for the educational attainment, memory, and RT phenotypes showed enrichment of central nervous system tissue types.

### Functional analysis and gene expression

Using the GTEx database (http://www.broadinstitute.org/gtex/), three cis-eQTL associations were identified for the 18 independent genome-wide significant SNPs found in the present report that were also included in this database (Supplementary Table S9). These were rs13086611, rs11130222 (an intronic SNP in *CAMKV*), and rs2142694 and they potentially regulate *FAM212A*, *RBM6* and *CYP2D6/SMDT1/NAGA*, respectively. For this study, data mining of regulatory elements was restricted to normal cell lines/tissues. There was evidence of regulatory elements associated with all 12 of the independent genome-wide significant SNPs included in the Regulome DB database. (http://www.regulomedb.org/)(Supplementary Table S9).

## Discussion

The results of the present study make novel contributions to three scientific aims of GWAS: helping towards identifying specific mechanisms of genomic variation; describing the genetic architecture of complex traits; and predicting phenotypic variation in independent samples. The most important novel contribution of the present study is the discovery of many new genome-wide significant genetic variants associated with reasoning ability, cognitive processing speed and the attainment of a college or university degree. The study provided robust estimates of the SNP-based heritability of the four cognitive variables and their genetic correlations. The study makes important steps toward genetic consilience, because several of the genomic regions identified by the present analyses have previously been associated in GWASs of general cognitive function, executive function, educational attainment, intracranial volume, neurodegenerative disorders and Alzheimer's disease. The study was successful in using the GWAS results from UK Biobank to predict cognitive variation in new samples.

The present study identified some novel genes/loci associated with individual differences in cognitive functions. These include, for RT, a locus on chromosome 12q24 containing the growth repressor *SH2B3*, and the RNA processing factor *ATXN2*, previously associated with a large number of disease susceptibilities including: type 1 diabetes, multiple sclerosis, spinocerebellar ataxia type 2, Parkinson's disease, longevity and many more.^52^ Phenotypically, processing speed and longevity are associated.^63^ Therefore, it is possible that this locus is influencing RT through its influence on a number of health traits. The most significant finding for verbal–numerical reasoning was for a gene-dense region on chromosome 22, and replication of this region was observed with general cognitive function. This region contains the cytochrome P450 gene (*CYP2D6*), which uses hydroxytryptamines (such as serotonin) and neurosteroids as endogenous substrates^64^ and may explain some of the links between cognitive functions and mental illnesses. Given the associations of this region with drug metabolism, it would be interesting to investigate this finding further with specific reference to medication use and psychiatric disease history. Brain size and cognitive ability are correlated phenotypically,^65^ and the significant hit in the GWAS of intracranial volume^37^ was also significant in SNP- and gene-based analyses in the present study.

Previously published associations between a number of genes and cognitive function and educational attainment have been replicated. *CADM2* (chromosome 3), which was previously associated with executive functioning and processing speed^21^ and showed suggestive association with general cognitive function,^13^ was associated with educational attainment in this study. *CADM2* encodes a synaptic cell adhesion molecule and is important in maintaining synaptic circuitry of the central nervous system.^66^ The two genes linked previously to educational attainment and cognitive function^23^ and that were associated with verbal–numerical reasoning and memory in UK Biobank are associated with the regulation of post-synaptic *N*-methyl d-aspartate receptors (*NRXN1*) and neuronal differentiation (*POU3F2*). Genetic variation in the *N*-methyl d-aspartate receptor complex has been previously shown to have an enriched association with cognitive abilities.^67^ Several specific SNPs previously associated with general cognitive function^13^ were associated with educational attainment and verbal–numerical reasoning, with single hits associated with RT and memory phenotypes in UK Biobank. This suggests that educational attainment can be used to some extent as a proxy for cognitive function (particularly verbal–numerical reasoning) in this sample. The genes *ATXN2L* and *SH2B1* (both on chromosome 16), previously linked to duration of education,^24^ were associated with both educational attainment and verbal–numerical reasoning in UK Biobank, as were three SNPs identified by Rietveld *et al.*^23^ as being associated with education and cognitive function.

Evidence of regulatory elements associated with all 12 of the genome-wide significant independent SNPs included in the Regulome DB database was identified within normal tissues and cell lines. The regulatory elements identified include position weight matrix, transcription factor binding sites, histone modifications, DNase hypersensitive sites and FAIRE sites. This evidence suggests that the associated SNPs are in sites of active transcription and could have a regulatory role on transcription. Three of the SNPs are potentially eQTLs.

The SNP-based estimate of heritability for verbal–numerical reasoning (31%) was highly consistent with previous estimates based on a general cognitive ability phenotype that had been composed using three or more diverse cognitive tests.^13, 14, 18^ Using the summary GWAS data from the present study to predict cognitive variation in independent samples (Supplementary Table S4) produced the largest *R*^2^ values in this field to date, with sometimes over 5% of variance explained, especially in the more crystallized cognitive functions such as vocabulary. Previously, values of 1 to 2% have been typical.^13^

The present study has several strengths. It has the largest sample size to date for a GWAS of any cognitive phenotype. It has the added advantage of being a single sample rather than a patchwork of small GWASs requiring meta-analysis. The tests offered were the same for all UK Biobank participants, so the phenotypic heterogeneity of previous cognitive GWASs has been overcome. It has phenotypes covering three important cognitive domains and a measure of educational attainment that could be used as a proxy for cognitive function. All individuals were of white British ethnicity, which minimized population stratification. The genotyping and its QC were performed in a consistent way.

The pattern of the present study's results, whereby the educational attainment and verbal–numerical reasoning variables had higher heritability and more genome-wide significant hits than RT or memory, can be understood with reference to some regularities in the relevant literature and, of course, on time constraints placed on data collection in a study that has a wide-ranging health remit. First, general cognitive ability, or strong indicators of it, tend to be more heritable than specific cognitive functions such as processing speed and memory.^8, 10, 12, 68^ Second, tests of verbal ability and reasoning are among those tests that have higher loadings on the latent trait of general cognitive ability, and tests of memory and processing speed have lower loadings.^15, 69, 70, 71^ Third, the RT and memory tests in UK Biobank were handicapped further by being very brief. The RT test included a far smaller number of trials than is typical for large surveys in the UK, which have used 40 trials in choice RT procedures.^72, 73^ The memory test was based on the recall of a single 12-item matrix with six pairs of stimuli. This is both a brief and unusual type of test in the field of declarative memory; more is known about the psychometric characteristics and genetic foundations of declarative memory tests such as word list and paragraph recall.^20^ The test–retest correlation of the memory variable was particularly low (*r*=0.15). When compared with phenotypic correlations of similar tests in the Lothian Birth Cohort 1936, correlations for the UK Biobank tests are about or more than 0.1 lower (Supplementary Table S10). Fourth, recent evidence from previous large studies make the strong evidence of a genetic contribution to educational attainment in the UK Biobank sample unsurprising. Despite their often being used as social background/environmental variables,^68^ educational attainments are substantially heritable,^74^ with many of the same genes affecting different academic subjects,^75^ and they have high phenotypic and genetic correlations with cognitive ability test scores, especially general cognitive ability.^25, 68, 76^ Moreover, it has already been demonstrated that educational attainment is useful as a proxy variable for cognitive ability in GWAS analyses.^23^

This accumulating evidence is consistent with the interpretation that, to some extent, educational attainments are a product of genetic contributions to cognitive ability, but with two emphatic qualifications. First, it is obvious that there are other—especially social—determinants of whether or not people achieve certain educational outcomes.^68^ Second, there is evidence that the variation in educational attainments that is caused by genetic differences is shared with traits other than intelligence, such as personality dimensions.^75, 77^ Therefore, we predict that not all of the genome-wide significant hits associated with the attainment of a college or university degree in the present study will be associated with cognitive differences; some might be associated with personality and other heritable, educational relevant traits.

The present study has limitations. Although the sample size is large for its field, GWASs of other complex traits such as height demonstrate that even larger sample sizes are required, and that these will reveal many more significant genetic variants. Another limitation concerns the cognitive tests. The three measures used were non-standard, bespoke tests. They did show the expected positive correlations with one another (and with education), but these correlations were lower than is typically found in studies of cognitive batteries which have tests with more items. The limitations of the RT and memory tests were discussed above. The verbal–numerical reasoning test had only 13 items, some of which had floor effects, and it had modest internal consistency. With more in-depth, albeit time-demanding tests, we expect to find even more genetic variants linked to differences in cognitive ability.^78^ Indeed, the educational variable heralds this. The participants in the sample were all white British, which restricts the generalizability of the results, which require extending to other groups. We also note that there are likely to be other types of genetic variation contributing to cognitive differences in addition into the common SNP variations studied here.

When genetic data on the full ~500 000 UK Biobank sample becomes available it is certain that more genetic variants will be found that are relevant for cognitive variation. This would enable robust replication of our current results in addition to other useful analyses. For example, sex- and age-moderation of genetic determinants could be studied with high power. It is possible, for example, that, as we have found for *APOE* variation,^13^ some genetic variants will have stronger effects at some ages than others, or even no effects whatsoever at some ages. There are also plans to enhance the cognitive testing in UK Biobank, which will afford the genetic study of additional and more detailed cognitive tests.

## Conclusion

The present results make advances in, and encourage much further work on the genetic foundations of cognitive differences. Until recently, GWASs of cognitive functions had provided converging information about their polygenic architecture—especially via SNP-based heritability estimates—and modest power to predict cognitive phenotypes in independent studies. However, they were near-bereft of significant, replicable genetic variants that could be followed-up to understand why some people have more efficient cognitive functioning than others. This drought is ending; work can begin on the genetically driven biological mechanisms of cognitive differences and the biological foundations of the many associations between cognitive abilities and bio-medical, health and social variables.

## Figures and Tables

**Table 1:** The proportion of the phenotypic variance explained by common SNPs (h^2^) and test–retest correlations for the three cognitive tests and educational attainment in UK Biobank

*Phenotype*	N	*h*^*2*^	*S.e.m.*	*Test–retest* N	*Test–retest Pearson correlation*
Reaction time	94 857	0.11	0.006	20 188	0.54
Memory	95 332	0.05	0.006	19 872	0.15
Verbal–numerical reasoning	30 801	0.31	0.018	4 696	0.65
Educational attainment	94 548	0.21	0.006		

Abbreviation: SNP, single-nucleotide polymorphism.

**Table 2:** Descriptive statistics and phenotypic (below diagonal) and genetic (above diagonal) correlations for the UK Biobank cognitive and educational variables in all genotyped participants

*Variable*	*Mean (s.d.)*	*Genetic (above)/phenotypic (below)/correlations*			
		*Reaction time*	*Memory*	*Verbal–numerical reasoning*	*Educational attainment*
Reaction time (ms)	555.08 (112.69)	—	0.179 (0.06)*	0.206 (0.05)*	0.066 (0.04)
Memory (errors)	4.06 (3.23)	0.116 (0.003)*	—	0.437 (0.06)*	0.126 (0.05)^†^
Verbal–numerical reasoning (maximum score 13)	6.16 (2.10)	0.156 (0.005)*	0.176 (0.005)*	—	0.729 (0.04)*
Educational attainment	30.5% With degree	0.099 (0.003)*	0.052 (0.003)*	0.338 (0.005)*	—

Genetic correlations are based on the results of genome-wide association studies of the UK Biobank variables. S.e.m. for the correlations are shown in parentheses. For the phenotypic variables, Pearson correlations were used for continuous–continuous correlations and point-biserial correlations for continuous-categorical correlations. All variables are coded such that higher scores indicate better performance. *indicates *P*-value<0.0001; ^†^indicates *P*-value<0.05. This table has been published previously.^62^
