# Supplementary material for: Genome-wide association study of cognitive functions and educational attainment in UK Biobank (N=112 151)
Source: Mol Psychiatry. 2016 Apr 5;21(6):758–67. doi: 10.1038/mp.2016.45 (PMC4879186; doi:10.1038/mp.2016.45)
Supplement: Supplementary Figure 1 [file mp201645x1.pdf]

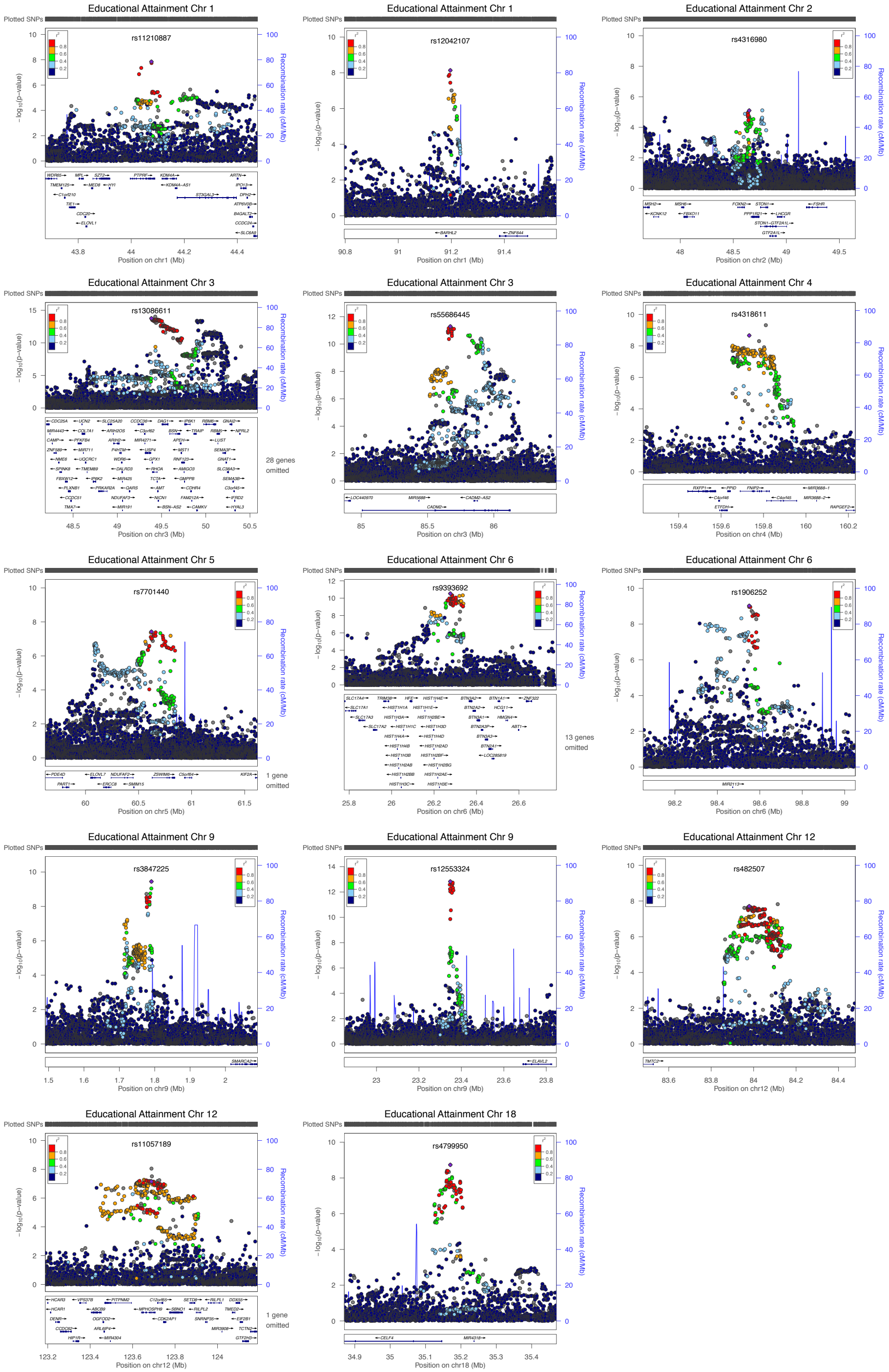
Educational Attainment Chr 1
rs11210887
Position on chr1 (Mb)
Educational Attainment Chr 1
rs12042107
Position on chr1 (Mb)
Educational Attainment Chr 2
rs4316980
Position on chr2 (Mb)
Educational Attainment Chr 3
rs13086611
28 genes omitted
Position on chr3 (Mb)
Educational Attainment Chr 3
rs55686445
Position on chr3 (Mb)
Educational Attainment Chr 4
rs4318611
Position on chr4 (Mb)
Educational Attainment Chr 5
rs7701440
1 gene omitted
Position on chr5 (Mb)
Educational Attainment Chr 6
rs9393692
13 genes omitted
Position on chr6 (Mb)
Educational Attainment Chr 6
rs1906252
Position on chr6 (Mb)
Educational Attainment Chr 9
rs3847225
Position on chr9 (Mb)
Educational Attainment Chr 9
rs12553324
Position on chr9 (Mb)
Educational Attainment Chr 12
rs482507
Position on chr12 (Mb)
Educational Attainment Chr 12
rs11057189
1 gene omitted
Position on chr12 (Mb)
Educational Attainment Chr 18
rs4799950
Position on chr18 (Mb)
Plotted SNPs
Recombination rate (cM/Mb)
