# Supplementary figures and images for: Genome-wide association study of cognitive functions and educational attainment in UK Biobank (N=112 151)
Source: Mol Psychiatry. 2016 Apr 5;21(6):758–67. doi: 10.1038/mp.2016.45 (PMC4879186; doi:10.1038/mp.2016.45)

# Verbal-numerical Reasoning Chr 7

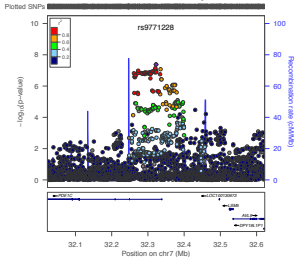

# Verbal-numerical Reasoning Chr 22

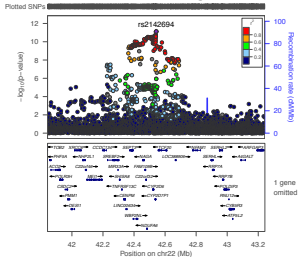

Supplement: Supplementary Figure 2 [file mp201645x2.pdf]

Reaction Time Chr 2

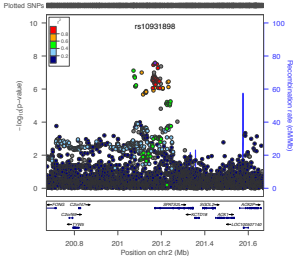

Reaction Time Chr 12

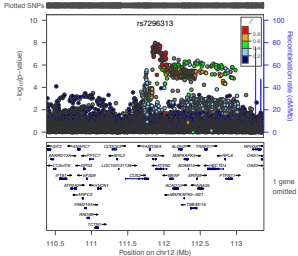

Supplement: Supplementary Figure 3 [file mp201645x3.pdf]
