# Supplementary material for: Genome-wide association study of cognitive functions and educational attainment in UK Biobank (N=112 151)
Source: Mol Psychiatry. 2016 Apr 5;21(6):758–67. doi: 10.1038/mp.2016.45 (PMC4879186; doi:10.1038/mp.2016.45)
Supplement: Supplementary Information [file mp201645x11.doc]

**Supplementary Materials**

**This document contains supplementary material for: Davies et al. Genome-wide association study of cognitive functions in UK Biobank (N = 112 151)**

*1. Supplementary Methods*

*2. References*

*3. Supplementary Tables*

Table S1. Descriptive statistics for the demographic variables and cognitive tests in UK Biobank.

Table S2A-C. Genome-wide significant SNP-based association results (*P*<5 x 10^-8^)

Table S3A-D. Genome-wide significant gene-based hits (*P*<2.8 x 10^-6^).

Table S4A-B. Polygenic prediction of cognitive traits in the Lothian Birth Cohort 1936 and Generation Scotland.

Table S5A-C. SNP look-ups of UK Biobank top hits in previous GWAS meta-analyses.

Table S6A-E. SNP look-ups in UK Biobank for the top hits from previous GWAS meta-analyses.

Table S7A-D. Gene look-ups for the top hits in previous GWAS meta-analyses.

Table S8. DEPICT gene prioritisation results.

Table S9. Functional annotation of the independent genome-wide significant SNPs.

Table S10. Phenotypic correlations for cognitive and educational variables in UK Biobank and Lothian Birth Cohort 1936.

*4. Supplementary Figures*

Figure S1 A-C. Regional association plots.

**Supplementary Methods**

**Genetic analyses: DEPICT**

We conducted three analyses using Data-driven Expression Prioritized Integration for Complex traits (DEPICT).^1^ Firstly, we prioritised genes in associated loci based on their biological relevance. Secondly we sought to identify the biological systems that they were a part of and, thirdly, we examined the tissue type in which these genes were most expressed.

DEPICT performs gene-set enrichment analysis by examining if independent associated loci show enrichment for reconstituted gene sets. This reconstitution is achieved by identifying genes that are co-regulated with other genes in the gene sets based on the results of a panel including 77 840 gene expression microarrays. If additional genes are found to be co-regulated with the genes from the original set, these additional genes are then added to the set.

Gene sets were included from multiple sources including, Gene Ontology^2^, Kyoto Encyclopaedia of genes and genomes^3^, and REACTOME^4^, the InWeb database^5^, and The Mouse Genetics initiative.^6^ Only gene sets with between 10 and 500 genes were included leading to a total of 14 461 gene sets.

The DEPICT analyses was performed using the scripts taken from GitHub (<https://github.com/DEPICTdevelopers>). In each of the cognitive phenotypes from UK Biobank, associated loci were defined as those where SNPs with a *P*-value of less than 1 x 10^-5^, and termed lead SNPs. Next SNPs were included in the associated loci if they were within 500kb and were in linkage disequilibrium (LD) of r^2^>0.1 with the lead SNP. In order to quantify linkage disequilibrium (LD), data from the 1000 Genomes Project Phase 1 CEU, GBR, TSI was used,^7^ was included, and SNP and gene positions were mapped using the GRCh37. These associated loci were created in PLINK^8,9^ using the clump function and used as input for DEPICT.

DEPICT prioritises genes and gene sets based on the predicted function of genes.^10^ Predicted gene function is derived using the results of 77 840 (two human, one rat and one mouse) microarray experiments each measuring the expression of 19 997 genes. These microarrays data were then re-normalised, before a probe by probe correlation matrix was derived. Next, within each of the four correlation matrices principal components analysis (PCA) was performed leading to the retention of 777 and 377 eigenvectors from the human microarray experiments and 677 and 375 microarray experiments eigenvectors (termed transcriptional components or TCs) from the mouse and rat experiments respectively. These were then converted to human homologues using the Ensembl database orthology mapping. The loading of each gene onto each of the TCs were then used to construct a gene-TC matrix. The gene-TC matrix is used to make predictions regarding the genes function across the predefined gene sets.

This is done by first deriving a transcriptional component for each of the gene sets by using a Welch’s t-test to determine if the TC loadings for each gene within a set deviated significantly from the TC loading of all the other gene sets. In order to derive the prediction about the genes function, each gene’s TC loading was correlated with the z score from the Welch’s t-test for each gene set in order to quantify each gene’s probability of being part of a given gene-set. Finally, these correlation *P*-values were converted to z-scores to create a gene – gene set matrix for use with gene prioritization and gene set analysis.

*Gene prioritisation*

The independent loci formed by the PLINK clumping procedure may encompass multiple genes. DEPICT exploits the fact that genes involved in a phenotype will have a tendency to be co-expressed and share similar annotations. This means that genes within associated loci that are more functionally similar to the other genes in associated loci are more likely to play a functional role. Three steps underlie the gene prioritisation procedure. Firstly, a metric is derived that quantifies the similarity of each gene to other genes in associated loci. This is derived by correlating the reconstituted gene set membership of a gene in an associated loci against that of the other genes across all associated loci. This is performed for each of the 14 461 gene sets. Next, the gene’s similarity score is normalised based on the distribution of a given gene’s similarity score using 1 000 gene-density matched loci obtained from 200 null GWAS. This stage corrects for the bias introduced by gene length and gene density. Thirdly, FDR is derived by repeating the scoring and normalisation steps 20 times using the top SNPs from the precomputed null GWAS. The FDR here is defined as the number of times the observed gene had a *P*-value equal or smaller than the 20 null GWAS, divided by the rank of the observed gene in the observed data.

*Gene-set analysis*

Using DEPICT enrichment of a gene set can be quantified by summing the gene set membership z scores for all genes within an associated locus and then summing across all loci. Next, random loci are created that are matched for gene density and a z score is computed for the random loci. This is performed 1000 times. The observed z score is then adjusted by subtracting the mean of the 1000 z scores from the random loci before being divided by their standard deviation. This is then converted into a *P*-value. In order to derive an experiment-wise FDR the first two steps were repeated 20 times. The aim of this analysis is to determine if the genes in the associated loci share biological functions, as genes which are driving the association are more likely to be causal candidates should they converge on a particular mechanism.^5,11,12^

*Tissue and cell analysis*

DEPICT contains the results of 37 427 human microarray experiments which can be incorporated into the analysis pipeline to determine if the genes in the associated loci are highly expressed in the 209 medical subject heading tissue and cell annotations. The method used to determine enrichment of a specific tissue or cell lines is as described above for gene set analysis.

**Functional annotation and gene expression**

For the 20 independent genome-wide significant SNPs identified by LD clumping, evidence of expression quantitative trait loci (eQTL) and functional annotation were explored using publicly available online resources. The Genotype-Tissue Expression Portal (GTEx) ([http://www.gtexportal.org](http://www.gtexportal.org/)) was used to identify eQTLs associated with the SNPs. Functional annotation was investigated using the Regulome DB database^13^ (<http://www.regulomedb.org/>. Regulome DB was used to identify regulatory DNA elements in non-coding and intergenic regions of the genome in normal cell lines/tissues.

**Cognitive phenotypes for polygenic prediction analysis**

The following section provides brief descriptions and references for the cognitive phenotypes we predicted in the Lothian Birth Cohort 1936 and Generation Scotland using the polygenic profile scores derived from our UK Biobank GWAS analysis. Full results for the polygenic prediction can be found in Supplementary Table S4.

*Lothian Birth Cohort 1936 (LBC1936)*

For the LBC1936 sample, we used the polygenic scores to predict three broad factors of cognitive ability. All were calculated as the first, unrotated principal component extracted from multiple tests, completed by the vast majority of the 1 091-strong LBC1936 sample at mean age 69.53 years (SD = 0.83). First, ‘Fluid cognitive ability’ was indicated by six tests taken from the Wechsler Adult Intelligence Test, 3^rd^ UK Edition (WAIS-III^UK^)^14^: Symbol Search, Digit-Symbol Substitution, Matrix Reasoning, Block Design, Letter-Number Sequencing, and Digit Span Backward. Second, ‘Memory’ was indicated by four tests from the Wechsler Memory Scale, 3^rd^ UK Edition (WMS-III^UK^)^15^: Logical Memory, Verbal Paired Associates, and Spatial Span, as well as the Digit Span Backward and Letter-Number Sequencing tests from the WAIS-III^UK^.^14^ Third, ‘Processing Speed’ was indicated by five tests: Symbol Search and Digit-Symbol Substitution from the WAIS-III^UK^, along with a computer-based test of visual Inspection Time^16^, and tests of Simple and 4-Choice Reaction Time presented on a dedicated instrument.^17^

In addition, we tested the association of the polygenic profile scores with cognitive ability in childhood (age 11) as measured by the Moray House Test No. 2,^18^ the same test taken at age 70,^19^ and the change between age 11 and 70 in this test (the latter being an index of cognitive change across the lifespan). Finally, the sample were all tested at age 70 on the National Adult Reading Test (NART), a vocabulary-based test that is widely used as an indicator of peak prior cognitive function.^20^

*Generation Scotland (GS)*

For the GS sample, we analysed the association of the polygenic profile score with four individual cognitive tests as well as two principal components derived from them. The four tests were Digit-Symbol Substitution from the WAIS-III^UK^,^14^ Logical Memory from the WMS-III^UK^,^15^ Verbal Fluency on the letters C, F, and L,^21^ and the Mill Hill Vocabulary Scale (with junior and senior synonyms combined).^22^ We extracted a ‘fluid’ general factor (‘fluid *g*’) from the first three tests (that is, not including the vocabulary test) and also an ‘overall’ general factor (‘*g*’) from all four tests. As for the Lothian Birth Cohort 1936 sample, both were the first unrotated principal component.

**SNP look-ups of UK Biobank hits in CHARGE general cognitive function GWAS**

For SNP look-ups in the CHARGE general cognitive function GWAS^23^ only a subset of the published data was available due to individual cohort restrictions on data usage (N = 36 840). The cohorts included are listed below:

The Austrian Stroke Prevention Study

Berlin Aging Study II

CROATIA-Split Study

CROATIA-Korčula Study

Erasmus Rucphen Study

Generation Scotland

Helsinki Birth Cohort Study

Health and Retirement Study

Hunter Community Study

Lothian Birth Cohort 1921

Lothian Birth Cohort 1936

The Rush Memory and Aging Project

Norwegian Cognitive NeuroGenetics Cohort

The Older Australian Twins Study

Orkney Complex Disease Study

PROspective Study of Pravastatin in the Elderly at Risk

The Religious Orders Study

The Rotterdam Study

The Sydney Memory and Aging Study

Tasmanian Study of Cognition and Gait

Three City Study

**Supplementary References**

1. Pers TH, Karjalainen JM, Chan Y, Westra HJ, Wood AR, Yang J *et al*. Biological interpretation of genome-wide association studies using predicted gene functions. *Nat Commun* 2015; **6**: (5890).

2. Ashburner M, Ball CA, Blake JA, Botstein D, Butler H, Cherry JM *et al*. Gene Ontology: tool for the unification of biology. *Nat Genet* 2000; **25**: 25-29.

3. Kanehisa M, Goto S, Sato Y, Furumichi M, Tanabe M. KEGG for integration and interpretation of large-scale molecular data sets. *Nucleic Acids Res* 2011; **40**: D109–D114.

4. Croft D, Mundo AF, Haw R, Milacic M, Weiser J, Wu G *et al*. The Reactome pathway knowledgebase. *Nucleic Acids Res* 2014; **42**: D472-D477.

5. Lage K, Karlberg EO, Størling ZM, Olason PI, Pedersen AG, Rigina O *et al*. A human phenome-interactome network of protein complexes implicated in genetic disorders. *Nat Biotechnol* 2007; **25**: 309-316.

6. Blake JA, Bult CJ, Eppig JT, Kadin JA, Richardson JE, Mouse Genome Database Group. The Mouse Genome Database: integration of and access to knowledge about the laboratory mouse. *Nucleic Acids Res* 2013; **42**: D810–D817.

# 7. The 1000 Genomes Project, An integrated map of genetic variation from 1,092 human genomes. *Nature* 2012; 491: 56-65.

8. Purcell S. PLINK v1.07. http://pngu.mgh.harvard.edu/purcell/plink/

9. Purcell S, Neale B, Todd-Brown K, Thomas L, Ferreira MAR, Bender D, *et al.* PLINK: a toolset for whole-genome association and population-based linkage analysis. *Am J Hum Genet* 2007; **81(3)**: 559-575.

10. Fehrmann RS, Karjalainen JM, Krajewska M, Westra HJ, Maloney D, Simeonov A *et al*. Gene expression analysis identifies global gene dosage sensitivity in cancer. *Nat Genet* 2015; **47**: 115-125.

11. Franke L, Van Bakel H, Fokkens L, De Jong ED, Egmont-Petersen M, Wijmenga C. Reconstruction of a functional human gene network, with an application for prioritizing positional candidate genes. *Am J Hum Genet* 2006; **78**: 1011-1025.

12. Raychaudhuri S, Plenge RM, Rossin EJ, Ng ACY, International Schizophrenia Consortium, Purcell SM *et al*. Identifying relationships among genomic disease regions: predicting genes at pathogenic SNP associations and rare deletions. *PLoS Genet* 2009; **5**: e1000534.

13. Boyle AP, Hong EL, Hariharan M, Cheng Y, Schaub MA, Kasowski M, *et al*. Annotation of functional variation in personal genomes using RegulomeDB. *Genome Res* 2012; **22(9)**: 1790-1797.

14. Wechsler D. *WAIS-III^UK^ Administration and Scoring Manual*. Psychological Corporation: London, UK, 1998.

15. Wechsler D. *WMS-III^UK^ Administration and Scoring Manual*. Psychological Corporation: London, UK, 1998.

16. Deary IJ, Simonotto E, Meyer M, Marshall A, Marshall I, Goddard N, *et al.* The functional anatomy of inspection time: an event-related fMRI study. *Neuroimage* 2004; **22**: 1466–1479.

17. Deary IJ, Der G, Ford G. Reaction times and intelligence differences: A population-based cohort study. *Intelligence* 2001; **29**: 389–399.

18. Scottish Council for Research in Education. *The Trend of Scottish Intelligence*. University of London Press: London, UK, 1949.

19. Deary IJ, Whiteman MC, Starr JM, Whalley LJ, Fox HC. The impact of childhood intelligence on later life: following up the Scottish Mental Surveys of 1932 and 1947. *J Pers Soc Psychol* 2004; **86**: 130–147.

20. Nelson HE, Willison JR. *National Adult Reading Test (NART) Test Manual*, 2nd edn. NFER-Nelson: Windsor, UK, 1991.

21. Lezak MD, Howieson DB, Loring DW. *Neuropsychological Assessment*, 4th edn. Oxford University Press: Oxford, UK, 2004.

22. Raven JC, Court JH, Raven J. *Manual for Raven's Progressive Matrices and Vocabulary Scales*. HK Lewis: London, UK, 1977.

23. Davies G, Armstrong N, Bis JC, Bressler J, Chouraki V, Giddaluru S et al. Genetic contributions to variation in general cognitive function: a meta-analysis of genome-wide association studies in the CHARGE consortium (N=53,949). *Mol Psychiatr* 2015; **20**: 183-192.

**Supplementary Tables**

**Table S1. Descriptive statistics for the demographic variables and cognitive tests in UK Biobank.**

|  | Variable | *N* | Mean | SD |
| --- | --- | --- | --- | --- |
| Demographics | Age (years) | 112 151 | 56.91 | 7.93 |
|  | Sex | 58 914 (52.5%) female; 53 237 (47.5%) male | - | - |
|  | College or University degree | 77 262 (69.5%) no;  33 852 (30.5%) yes | - | - |
| Cognitive tests | Verbal-numerical Reasoning (max. score = 13) | 36 035 | 6.15 | 2.10 |
|  | Reaction Time  (ms) | 111 483 | 555.08 | 112.68 |
|  | Memory  (no. of errors) | 112 067 | 4.06 | 3.23 |

**Table S2A.** Genome-wide significant SNP-based association results for Educational Attainment (P < 5 x 10^-8^). The results are ordered by significance of the association. The independent SNP signals, as determined by the LD Clumping analysis, are highlighted in red.

**Table S2B.** Genome-wide significant SNP-based association results for Verbal-numerical Reasoning (P < 5 x 10^-8^). The results are ordered by significance of the association. The independent SNP signals, as determined by the LD Clumping analysis, are highlighted in red.

**Table S2C.** Genome-wide significant SNP-based association results for Reaction Time (P < 5 x 10^-8^). The results are ordered by significance of the association. The independent SNP signals, as determined by the LD Clumping analysis, are highlighted in red.

*(See attached Excel spreadsheet)*

**Table S3A.** Genome-wide significant gene-based hits (P < 2.8 x 10^-6^) in the MAGMA gene-based analysis for Educational Attainment. NSNPS is the number of SNPs in the gene; Effect Size is the number of independent SNPs in the gene.

**Table S3B**. Genome-wide significant gene-based hits (P < 2.8 x 10^-6^) in the MAGMA gene-based analysis for Verbal-numerical Reasoning. NSNPS is the number of SNPs in the gene; Effect Size is the number of independent SNPs in the gene.

**Table S3C.** Genome-wide significant gene-based hits (P < 2.8 x 10^-6^) in the MAGMA gene-based analysis for Reaction Time. NSNPS is the number of SNPs in the gene; Effect Size is the number of independent SNPs in the gene.

**Table S3D.** Genome-wide significant gene-based hits (P < 2.8 x 10^-6^) in the MAGMA gene-based analysis for Memory. NSNPS is the number of SNPs in the gene; Effect Size is the number of independent SNPs in the gene.

*(See attached Excel spreadsheet)*

**Table S4A.** Polygenic prediction of cognitive traits in the Lothian Birth Cohort 1936. Phenotypic correlations are also shown.

**Table S4B**. Polygenic prediction of cognitive traits in Generation Scotland. Phenotypic correlations are also shown.

See Supplementary Methods for descriptions of the cognitive traits.

*(See attached Excel spreadsheet)*

**Table S5A**. SNP look-ups of genome-wide significant association results for UK Biobank Educational Attainment (P<5 x 10^-8^) in general cognitive function (CHARGE; Davies et al., 2015), SSGAC educational attainment (years and college; SSGAC; Rietveld et al., 2013) and childhood intelligence (CHIC; Benyamin et al., 2014). The results are ordered by significance of the association in the UK Biobank Educational Attainment GWAS.

**Table S5B.** SNP look-ups of genome-wide significant association results for Verbal-numerical reasoning (P<5 x 10^-8^) in general cognitive function (CHARGE; Davies et al., 2015), SSGAC educational attainment (years and college; SSGAC; Rietveld et al., 2013) and childhood intelligence (CHIC; Benyamin et al., 2014). The results are ordered by significance of the association in the Verbal-numerical Reasoning GWAS.

**Table S5C**. SNP look-ups of genome-wide significant association results for Reaction Time (P<5 x 10^-8^) in general cognitive function (CHARGE; Davies et al., 2015), SSGAC educational attainment (years and college; SSGAC; Rietveld et al., 2013) and childhood intelligence (CHIC; Benyamin et al., 2014). The results are ordered by significance of the association in the Reaction Time GWAS.

*(See attached Excel spreadsheet)*

**Table S6A.** SNP look-ups for genome-wide significant hits in GWAS of general cognitive function (CHARGE; Davies et al., 2015).

**Table S6B**. SNP look-ups for genome-wide significant hits in GWAS of memory (CHARGE; Debette et al., 2015).

**Table S6C**. SNP look-ups for genome-wide significant hits in GWAS of education (SSGAC; Rietveld et al., 2013, 2014).

**Table S6D**. SNP look-ups for genome-wide significant hits in GWAS of Alzheimer’s disease (IGAP; Lambert et al., 2013).

**Table S6E**. SNP look-ups for genome-wide significant hits in GWAS of intracranial volume (ENIGMA; Hibar et al., 2015).

*(See attached Excel spreadsheet)*

**Table S7A.** Look-ups for genome-wide significant gene-based hits in GWAS meta-analyses of general cognitive function (CHARGE; Davies et al., 2015).

**Table S7B.** Look-ups for genome-wide significant gene-based hits in GWAS meta-analyses of education (college degree) (SSGAC; Rietveld et al., 2013).

**Table S7C.** Look-ups for genome-wide significant gene-based hits in GWAS meta-analyses of education (years of education) (SSGAC; Rietveld et al., 2013).

**Table S7D.** Look-ups for genome-wide significant gene-based hits in GWAS meta-analyses of childhood cognitive function (CHIC; Benyamin et al., 2014).

*(See attached Excel spreadsheet)*

**Table S8.** DEPICT gene prioritisation results. Genes prioritised by DEPICT for educational attainment that withstood correction for multiple comparison. Top cis eQTL indicates SNPs within the associated loci that show evidence for eQTLs in whole blood (Westra et al., 2013).

*(See attached Excel spreadsheet)*

**Table S9. Functional annotation of the independent genome-wide significant SNPs. All information contained in this table was extracted from the GTEx database (**[**http://www.broadinstitute.org/gtex/**](http://www.broadinstitute.org/gtex/)**) and the Regulome DB database (**[**http://regulome.stanford.edu/index**](http://regulome.stanford.edu/index)**).**

|  |  | **cis-eQTL** | **Position weight matrix** | **Transcription factor binding site** | **Histone modifications** | **DNase hypersensitive sites** | **FAIRE sites** | **DMR** |
| --- | --- | --- | --- | --- | --- | --- | --- | --- |
|  | **Educational Attainment** |  |  |  |  |  |  |  |
|  | rs13086611 | y | y | n | y | n | n | n |
|  | rs11130222 | y | y | y | y | y | n | n |
|  | rs12553324 | n | y | n | y | n | n | n |
|  | rs55686445 | n | - | - | - | - | - | - |
|  | rs9393692 | n | y | n | y | n | n | n |
|  | 2:48696432_G_A | - | - | - | - | - | - | - |
|  | rs3847225 | n | - | - | - | - | - | - |
|  | rs1906252 | n | - | - | - | - | - | - |
|  | rs4799950 | n | - | - | - | - | - | - |
|  | rs4318611 | n | y | n | y | n | n | n |
|  | rs112374913 | n | - | - | - | - | - | - |
|  | rs12042107 | n | y | y | y | y | n | n |
|  | rs11210887 | n | y | n | y | n | n | n |
|  | rs482507 | n | y | n | y | n | n | n |
|  | rs7701440 | n | y | n | y | n | n | n |
|  | **Verbal-numerical**  **Reasoning** | |  |  |  |  |  |  |
|  | rs2142694 | y | y | n | y | n | n | n |
|  | 14:66113725_C_A | - | - | - | - | - | - | - |
|  | rs9771228 | n | - | - | - | - | - | - |
|  | **Reaction Time** |  |  |  |  |  |  |  |
|  | rs7296313 | n | y | n | y | n | n | n |
|  | rs10931898 | n | y | y | y | y | y | n |

**Table S10.** Phenotypic correlations for cognitive and educational variables in UK Biobank (below diagonal) (Reaction Time, Memory, Verbal-numerical Reasoning, Educational Attainment) and Lothian Birth Cohort 1936 (above diagonal) (Logical Memory immediate, 4-choice reaction time, NART, Educational Attainment).

Standard errors in parentheses. All correlations significant at p < .0001.

|  | **Reaction Time** | **Memory** | **Crystallized ability** | **Educational Attainment** |
| --- | --- | --- | --- | --- |
| **Reaction Time** | - | -0.241 (0.033) | -0.239 (0.033) | -0.121 (0.034) |
| **Memory** | 0.116 (0.003) | - | 0.384 (0.031) | 0.232 (0.033) |
| **Crystallized ability** | 0.156 (0.005) | 0.176 (0.005) | - | 0.523 (0.029) |
| **Educational Attainment** | 0.099 (0.003) | 0.052 (0.003) | 0.338 (0.005) | - |

**Figure S1.** Regional association plots of genomic regions that demonstrated genome-wide significance (P<5 x 10^-8^) in the SNP-based association analyses for: (A) Educational Attainment, (B) Verbal-numerical Reasoning, and (C) Reaction Time. The circles represent individual SNPs, with the colour indicating pairwise linkage disequilibrium (LD) to the SNP indicated by the purple diamond (calculated from 1000 Genomes Nov 2014 EUR). The purple diamond indicates the most significant SNP for which LD information was available in the 1000G reference sample. The solid blue line indicates the recombination rate and –log10 P-values are shown on the y-axis.

*(See attached PDF)*
